# The molecular mechanism of cytoadherence to placental or tumor cells through VAR2CSA from *Plasmodium falciparum*

**DOI:** 10.1038/s41421-021-00324-8

**Published:** 2021-10-19

**Authors:** Weiwei Wang, Zhaoning Wang, Xiuna Yang, Yan Gao, Xiang Zhang, Long Cao, Aguang Dai, Jin Sun, Lei Sun, Lubin Jiang, Zhenguo Chen, Lanfeng Wang

**Affiliations:** 1 The Center for Microbes, Development and Health, CAS Key Laboratory of Molecular Virology & Immunology, Institut Pasteur of Shanghai, Chinese Academy of Sciences, University of Chinese Academy of Sciences, Shanghai, China; 2 College of Life Sciences, University of Chinese Academy of Sciences, Beijing, China; 3 Institute for Advanced Immunochemical Studies and School of Life Science and Technology, ShanghaiTech University, Shanghai, China; 4 Shanghai Fifth People’s Hospital, Fudan University, and Shanghai Key Laboratory of Medical Epigenetics, International Co-laboratory of Medical Epigenetics and Metabolism (Ministry of Science and Technology), Institutes of Biomedical Sciences, Fudan University, Shanghai, China

**Keywords:** Cryoelectron microscopy, Mechanisms of disease

Dear Ed﻿itor,

Pregnancy-associated malaria (PAM) threatened more than one million women and their infants in endemic regions in 2019. This resulted in maternal anemia, stillbirth, and infant death^1,2^. VAR2CSA encoded by a subfamily of var genes from *Plasmodium falciparum* (*P. falciparum*) named as var2csa, plays a vital role in the cytoadherence of infected erythrocytes to the placenta^3^. Chondroitin sulfate A (CSA), which is displayed mostly on the surface of placental or tumor cells, has been recognized as a specific ligand for VAR2CSA^4–7^. However, the molecular mechanism of cytoadherence to placental or tumor cells through VAR2CSA remains elusive.

In this study, the VAR2CSA ectodomain from *P. falciparum* strain 3D7 (~306 kDa, containing six Duffy-binding-like (DBL) domains, N-terminal sequence (NTS), and multiple inter-domains (IDs))^8^ (Fig. 1a) was recombinantly expressed and purified using the Sf9 insect cell secretory system. The cryo-EM structures of VAR2CSA ectodomain and its complex with CSA were determined at a resolution of 3.6 Å and 3.4 Å, respectively (Fig. 1b, c; Supplementary Figs. S1–S5 and Table S1). In line with the previously proposed model^9^, our structures showed that the core region is well-defined and covers NTS, DBL1X, DBL2X, ID2a, ID2b, DBL3X, ID3, and DBL4ε (Fig. 1b, c; Supplementary Fig. S6a). However, DBL5ε and DBL6ε formed a flexible wing region. Compared with the apo structure, the densities of the wing region in the complex were significantly improved, making it feasible for the flexible fitting of both DBL5ε and DBL6ε (Supplementary Fig. S4d). As for the core region, DBL2X and DBL4ε stacked closely with ID2a, ID2b, and ID3, and formed the most stable core center, which served as a base for anchoring DBL3X and DBL1X at the top or bottom sites, respectively (Fig. 1b, c). Intriguingly, a highly basic pocket formed by DBL2X, DBL1X, NTS, and DBL4ɛ has been identified to accommodate a dodecasaccharide with six sulfated disaccharide repeats from CSA, which fitted well into the density map. Moreover, 16 residues were identified to be responsible for the direct interaction with 10/12 monosaccharides, except for the 6th and 11th units of CSA dodecasaccharide. Among them, there are nine, four, and three residues derived from DBL2X, DBL4ɛ, and NTS, respectively. Furthermore, the nine residues of DBL2X were shown to interact directly with 7/10 monosaccharide units and thus may account for the major contribution to CSA binding (Fig. 1g; Supplementary Figs. S4e, f, S6b, c).

**Fig. 1:**
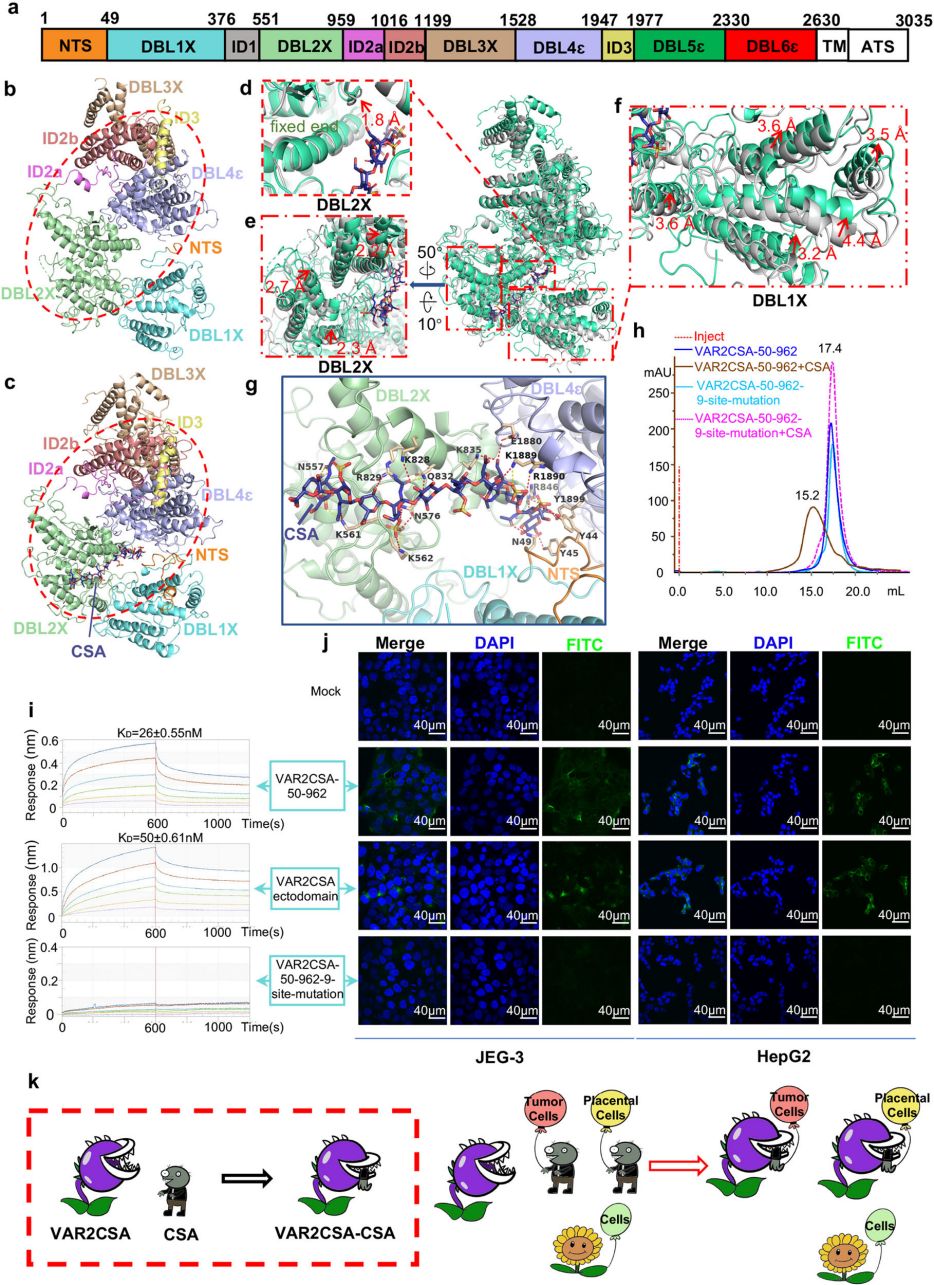
Overall structures of VAR2CSA ectodomain. **a** Schematic sequence architecture of VAR2CSA of *P. falciparum* 3D7 strain. Domains reconstructed according to the cryo-EM density map were colored respectively. NTS, orange; DBL1X, aquamarine; DBL2X, pale green; ID2a, violet; ID2b, salmon; DBL3X, wheat; DBL4ɛ, light blue; ID3, pale yellow; DBL5ɛ, green; DBL6ɛ, red; ID1, gray. Color codes were used throughout this study unless otherwise noted. **b** The ribbon diagram of apo VAR2CSA ectodomain core region. **c** The ribbon diagram of VAR2CSA-CSA core region. The core center was highlighted using red ovals in **b** and **c**. **d**–**f** Structural comparison between VAR2CSA-CSA with apo VAR2CSA ectodomain by aligning DBL4ɛ domains. The helix associated with CSA in DBL2X domain exhibited a 1.8 Å bend outward at the binding site while the distal end remained fixed (**d**). The surficial helixes in DBL2X moved about 2 Å toward the CSA binding pocket (**e**). The DBL1X domain went through a significant conformational change at a distance of more than 3.2 Å to close the CSA binding pocket (**f**). **g** Interactions between CSA and VAR2CSA were shown in the binding pocket. Interacting residues from NTS, DBL2X, and DBL4ɛ were highlighted individually. **h** Gel filtration chromatography of VAR2CSA-50-962 and VAR2CSA-50-962-9-site-mutation (N557D, K561E, K562E, N576D, K828E, R829E, Q832E, K835E, R846E) in the presence or absence of CSA on a Superose 6 increase 10/300 GL column. **i** The binding kinetics of VAR2CSA ectodomain, VAR2CSA-50-962, and VAR2CSA-50-962-9-site-mutation were further assessed using Octet RED 96, and equilibrium constant (*K*_D_) values were calculated accordingly. **j** The binding activities of VAR2CSA-50-962, VAR2CSA ectodomain, and VAR2CSA-50-962-9-site-mutation to JEG-3 cells or HepG2 cells were tested by immunofluorescence assay using confocal fluorescence microscopy, respectively. Green (stained with anti-His-FITC antibody) indicated VAR2CSA proteins; blue (DAPI) represented cell nuclei. **k** Model of VAR2CSA recognizing both placental cells and tumor cells. The cartoon exhibited the mechanism of VAR2CSA capturing the CSA ligand and showed the conformational change upon CSA binding in a red frame. The cartoon also illustrated the mechanism of VAR2CSA characteristically recognizing either placental cells or tumor cells by identifying the specific CSA displayed on the cell surface. VAR2CSA was drawn as cannibal plants in two states corresponding to CSA binding or not, respectively. CSA modifications were drawn as zombies and other glycol-modifications as sunflowers. Cells were depicted as balloons in different colors. Tumor cells, salmon; Placental cells, yellow; Normal cells, light green.

Owing to its high resolution and potential high stability in the core center, DBL4ɛ was used as an immobilized reference for the structural alignment between the VAR2CSA-CSA and VAR2CSA ectodomain (Fig. 1d–f). Interestingly, a significant conformational change in the core region was observed (Supplementary Movie S1). Firstly, the DBL1X moves closer to DBL4ɛ at more than 3.2 Å (ranging from 3.2 Å to 4.4 Å) for multi-helixes to facilitate NTS interaction with CSA and close the pocket (Fig. 1f). Secondly, there was a 1.8 Å outward bend for the key CSA binding helix of DBL2X in the deep pocket (Fig. 1d). Lastly, the rest of DBL2X moved about 2 Å inward to shrink the pocket (Fig. 1e).

Next, we aimed to identify the minimal structural elements required for CSA binding. First, the individual structures of four DBL domains (DBL1X, DBL2X, DBL3X, and DBL4ɛ) in the core region are quite similar to each other, with the typical “3 + 2” helix bundle shared (Supplementary Fig. S7). Sequence alignment showed that the nine key residues in DBL2X were rarely conserved within intramolecular DBL domains but highly conserved across various *P. falciparum* strains, suggesting a common CSA recognition mechanism dominated by DBL2X (Supplementary Figs. S8 and S9). Accordingly, two truncations, VAR2CSA-50-962 (DBL1X and DBL2X) and VAR2CSA-550-962 (DBL2X alone), were generated, and the CSA binding activity was evaluated using gel filtration chromatography. Similar to the VAR2CSA ectodomain, both VAR2CSA-50-962 and VAR2CSA-550-962 formed stable complexes with CSA (Supplementary Fig. S10). VAR2CSA-50-962 was selected as a representative for subsequent experiments due to its high yield. The nine key residues were mutated to D or E to obtain VAR2CSA-50-962 N557D, K561E, K562E, N576D, K828E, R829E, Q832E, K835E, and R846E (VAR2CSA-50-962-9-site-mutation).

Both VAR2CSA-50-962 and VAR2CSA-50-962-9-site-mutation in the presence or absence of CSA were subjected to analysis using gel filtration chromatography. Interestingly, the CSA binding activity of the VAR2CSA-50-962-9-site-mutation was totally disrupted in comparison with VAR2CSA-50-962 (Fig. 1h). Additionally, Octet RED 96 was used to test the CSA binding affinities of the VAR2CSA ectodomain, VAR2CSA-50-962, and VAR2CSA-50-962-9-site-mutation. In accordance with the results of chromatography above, the VAR2CSA ectodomain and VAR2CSA-50-962 exhibited a strong affinity for CSA, while the VAR2CSA-50-962-9-site-mutation showed no binding activity (Fig. 1i).

Significantly, it was further verified that the 9-site-mutation could eliminate the binding activity of VAR2CSA to placental cells or tumor cells using confocal fluorescence microscopy. Briefly, 500 nM individual protein (VAR2CSA ectodomain, VAR2CSA-50-962, or VAR2CSA-50-962-9-site-mutation) was incubated with cells (JEG-3 and HepG2 representing placental cells or tumor cells, respectively) pre-seeded on a slide. After thorough washing, the fluorescent-labeled anti-His monoclonal antibody was added to stain the retarded proteins, while DAPI was applied to identify cell nuclei. The slides were analyzed using an Olympus FV1200 laser scanning confocal microscope. Compared to the controls (no proteins added), VAR2CSA-50-962 and VAR2CSA ectodomain could specifically bind to JEG-3 cells and HepG2 cells, and no obvious difference in the binding affinity was observed between the two proteins, while the cell-binding activity of the VAR2CSA-50-962-9-site-mutation was eliminated (Fig. 1j). This is in line with the biochemistry results described above. Taken together, the 9-site-mutation in DBL2X can eliminate the binding activity of VAR2CSA to placental cells or tumor cells under the conditions tested.

Recently, the cryo-EM structures of the VAR2CSA ectodomain from *P. falciparum* strain FCR3 and VAR2CSA ectodomain from *P. falciparum* strain NF54 complexed with CSA were reported^10^. Meanwhile, another study also revealed the cryo-EM structures of the VAR2CSA ectodomain from *P. falciparum* strain FCR3 and its complex with placental chondroitin sulfate^11^. Uniquely, our structural study showed that CSA binding induced significant conformational changes to secure ligand binding, which was not reported by these two works^10,11^. The conformation in the former work^10^ is different from ours, which might be due to the fact that VAR2CSA proteins in apo structure and complex structure are from two different *P. falciparum* strains. In the other study^11^, FCR3 VAR2CSA shared a sequence identity of 79% with 3D7 VAR2CSA used in our study, but no ligand density was reported in their complex structure. Most importantly, beyond the structural study, we also generated VAR2CSA fragments and 9-site mutations, and evaluated their binding activity to either isolated CSA or placental and tumor cell lines. These results clearly showed that the 9-site-mutation in DBL2X eliminated the CSA binding activity in vitro and disrupted its binding to both placental and tumor cells.

In summary, we have elucidated the detailed molecular mechanism of cytoadherence to placental or tumor cells through VAR2CSA, presented in the working model (Fig. 1k). Our results may facilitate antigen design for the PAM vaccine development and improve the drug delivery systems targeting both placenta and tumor cells.

## Supplementary information

Supplementary Information

Supplementary Movie S1
