# Supplementary material for: The molecular mechanism of cytoadherence to placental or tumor cells through VAR2CSA from Plasmodium falciparum
Source: Cell Discov. 2021 Oct 19;7:94. doi: 10.1038/s41421-021-00324-8 (PMC8523661; doi:10.1038/s41421-021-00324-8)
Supplement: Supplementary file 1 — Supplementary Information [file 41421_2021_324_MOESM1_ESM.pdf]

Supplementary Information

Methods and Materials

VAR2CSA fragments expression and purification

VAR2CSA ectodomain coding sequence (residues 1-2630 from *P.falciparum* 3D7, GenBank ID: 811060) was synthesized with optimized codons for mammalian cell, and inserted into gp67-438-B vector with a N-terminal 6xHis Tag to prepare the bacmid and following recombinant baculovirus. The gp67-438-B modified from 438-B (Addgene) was used to express recombinant secretory proteins.

Sf9 cells were cultured in serum-free medium (SF900 II, Thermo Fisher) at 27°C with a speed of 130 rpm in a shaker (Yonglian). One liter of Sf9 cells (2×10^6/mL confluence) was infected with 5 mL P3 baculoviral stock obtained following the manufacturer's instructions (Invitrogen). The medium containing secreted protein was collected on the 4th day (about 72-hour post-infection) and centrifuged for 20 min at 6,700 g to remove cell pellets. The supernatant was precipitated by saturated ammonium sulfate. And the precipitate was acquired by centrifugation for 40 min at 12,000 g, and resuspended in buffer A (20mM MES, pH6.5). The sample was mixed with pre-equilibrated Ni-NTA Agarose beads (GE Healthcare) at a ratio of 2 mL beads per liter medium and stirred for 2 hours at 4°C. The slurry was loaded onto a 15-mL gravity column (Bio-Rad) and washed with wash buffer (20 mM MES pH 6.5, 500 mM NaCl, and 5% v/v glycerol) for ~30 column volumes. The beads were further washed using wash buffer supplemented with 10 mM imidazole until no trace of protein was detected in the flowthrough. VAR2CSA was eluted by wash buffer supplemented with 200 mM imidazole. It was then concentrated (Amicon Ultra-15 30,000 MWCO, Millipore) to ~500 μL and loaded onto a Superose6 increase 10/300 GL (GE Healthcare) equilibrated with buffer B (20 mM MES pH 6.5, 250 mM NaCl). The peak fractions containing final pure protein were collected, pooled, and concentrated to 3 mg/mL for the following experiments.

VAR2CSA-50-962 and VAR2CSA-550-962 were amplified and subcloned into the pET20b vector between NdeI and XhoI restriction sites with a C-terminal 6xHis tag. VAR2CSA-50-962-9-site-mutation was generated via the substitution of the synthesized fragment with 9 key residues

mutated (N557D, K561E, K562E, N576D, K828E, R829E, Q832E, K835E, and R846E). The above proteins were expressed in Rosetta-gamiB (DE3) of *E. coli.* (WEIDI Biotechnology). Briefly, when the recombinant bacteria grow to the OD value around 0.6 at 37°C, the temperature was lowered to 16°C for about half an hour. IPTG was then added to the culture at a final concentration of 0.2 mM. Cells were collected after 20-hour culture. Cell pellets were resuspended in wash buffer and lysed using Emulsiflex homogeniser (YongLian). The proteins were purified by sequential chromatography using Ni-NTA affinity, HiTrap S, gel filtration columns (GE Healthcare). According to the separation efficiency, Superose6 increase 10/300 GL was used for VAR2CSA-50-962 and VAR2CSA-50-962-9-site-mutation, and Superdex 75 10/300 GL for VAR2CSA-550-962.

CSA extracted from bovine trachea (Sigma, C9819) was used as the ligand for VAR2CSA. Various VAR2CSA proteins were incubated with CSA solution (10 mg/mL) overnight on ice and further separated by Superose6 increase 10/300 GL. The complex fractions were collected, pooled and concentrated to 3 mg/mL for the following experiments.

Cryo-samples preparation of VAR2CSA ectodomain

The VAR2CSA ectodomain was cross-linked and purified using Grafix[1]. Briefly, the glycerol gradient was prepared using light buffer (20 mM MES pH 6.5, 250 mM NaCl, 15% (v/v) glycerol) and heavy buffer (20 mM MES pH 6.5, 250 mM NaCl, 0.05% glutaraldehyde, 35% (v/v) glycerol). The samples were centrifuged at 38,000 rpm for 16 hours at 4°C using a Beckman SW41 Ti rotor. Subsequently, 200 μL per fraction were harvested and the cross-linking reaction was terminated by adding quench buffer (1 M Tris pH 6.5, 250 mM NaCl) to a final concentration of 40 mM Tris. Fractions containing cross-linked VAR2CSA monomer were pooled, concentrated, and dialyzed in buffer B. The cross-linked VAR2CSA monomer about 0.4 mg/mL was applied to the preparation of cryo-EM grids.

For negative staining EM analysis, carbon-coated copper grids were processed using a PELCO easiGlow (TED PELLA) cleaning system with a power of 30 W and a plasma current of 30 mA in the air for 30 s. Subsequently, samples (8 μL at a concentration of ~0.02 mg/mL) were applied

onto the grids above and stained twice using 2% (w/v) uranyl acetate solution at room temperature for the following examination via 200kV TEM (TF20, FEI).

As for the preparation of cryo-EM grids, Amorphous Alloy Film Au300 R1.2/1.3 grids (CryoMatrix) were processed in the H_2/O_2 mixture for 30 s using a Gatan 950 Solarus plasma cleaning system with a power of 5 W. Then cross-linked VAR2CSA ectodomain (3 μL at a concentration of ~0.4 mg/mL) was applied to the grids for instant incubation under a relative humidity of 100% at 4 °C. Next, the grids were blotted for 2 s with a blot force of 1 in a Vitrobot Mark IV (Thermo Fisher) and plunge-frozen in liquid ethane. Similarly, VAR2CSA-CSA (3 μL at a concentration of ~0.5 mg/mL) was blotted for 3 s with a blot force of -2 and plunge-frozen in liquid ethane.

Cryo-EM data collection and Image processing

The cryo-EM grids of cross-linked VAR2CSA ectodomain and VAR2CSA-CSA were firstly evaluated using a 200kV Talos Arctica microscope (Thermo Fisher). Cryo-EM datasets were collected on 300 kV Titan Krios microscope (Thermo Fisher) equipped with a Gatan K2 Summit direct electron detector and a 20-eV slit GIF Quantum energy filter (Gatan). The cryo-EM images were automatically recorded in the super-resolution counting mode using Serial-EM[2] software with a nominal magnification of 130,000 x (a super-resolution pixel size of 0.522 Å), and with a defocus ranging from -1.2 to -2.2 μm. Each micrograph stack was dose-fractionated into 36 frames with a total electron dose of ~ 57.6 $e^-/Å^2$ and a total exposure time of 7.2 s. Drift and beam-induced motion of the super-resolution movie stacks were corrected using MotionCor2[3] and binned twofold to a calibrated pixel size of 1.044 Å/pix, with both the dose weighted and non-dose weighted micrographs saved at the same time. The defocus values were estimated by Gctf[4] using the non-dose weighted micrographs. Other procedures of cryo-EM data processing were performed using RELION v3.0[5].

A total of 1,965 movies were recorded for the cross-linked VAR2CSA ectodomain. Among them, 1,800 micrographs were selected for further processing due to appropriate range of rlnDefocusU (5000-30000) and rlnCtfMaxResolution (2-6). Selected 2D class averages from 200kV cryo-EM data were low-pass filtered to 20 Å and used as references for auto-picking. Then an initial set of

984,517 particles were extracted for 2D classification. After several rounds of 2D classification, the relatively good classes with 960,039 particles were selected for three-dimensional reconstruction. Initially, one major class (~62% particles) was separated after 3D classification. To further improve the resolution, a second cycle of 3D classification with mask was carried out with 304,160 particles selected for 3D auto-refinement and postprocessing (the B-factor automatically estimated). The final resolution was evaluated according the gold-standard Fourier shell correlation (threshold = 0.143)[6]. Although the overall resolution is up to 3.6 Å, the density of the wing region (DBL5ε and DBL6ε) is quite weak due to high flexibility. The local refinement strategy for focused classification and refinement was carried out for the whole structure divided into two half parts. Consequently, the resolution of the core region could be improved to 3.1 Å by merging two local-refined maps. However, the map quality of the wing region remained unimproved, which made it hardly interpreted by *de novo* model building. The local resolution was evaluated by ResMap[7].

For VAR2CSA-CSA, 3,058 out of 3,356 micrographs were selected for further processing using similar procedures as above. Briefly, 1,163,625 auto-picked particles were extracted for 2D classification. After several rounds of 2D and 3D classification, 241,954 particles were selected for 3D auto-refinement and postprocessing. The final map was reconstructed at a resolution of 3.4 Å. Compared with apo structure, the density of the wing region has been significantly improved, which makes it feasible for the flexible fitting of both DBL5ε and DBL6ε. The resolution of the core region could be improved to 3.1 Å by merging local-refined maps.

Model building and refinement

The density map of VAR2CSA ectodomain was firstly interpreted using Phenix.map_to_model[8] to generate the initial model with the most helixes, which has been further improved by manual model building using COOT[9]. The structure of VAR2CSA-CSA was determined using the apo structure as a reference and manual model building in COOT with various refinement strategies. The structures have been further validated by validation module in Phenix (**Table S1**). All structural figures were prepared using UCSF Chimera[10], UCSF ChimeraX[11] or PyMOL[12].

Interaction evaluated using Octet RED 96

CSA were biotinylated and loaded onto SA sensor (Pall corporation). VAR2CSA fragments and mutant were then added for real-time association and dissociation analysis using Octet RED 96 (Fortebio) at room temperature. Data Analysis Octet was used for data processing.

Confocal fluorescence microscopy

Cells were pre-seeded on a slide, washed with PBS and fixed using 4% PFA/PBS on ice. After fixation, cells were blocked in 2.5% FBS for 30 min on ice and incubated with recombinant VAR2CSA proteins diluted in PBS with 0.25% FBS for 1 hour on ice. After washing with PBS by three times, the specimens were incubated with anti-His-FITC antibody (1:500) for 1 hour at room temperature in the dark and washed as described before. DAPI was applied to stain and locate the cell nuclei. Slides were further analyzed using a confocal microscope. Negative controls (Mock) were prepared with the same procedures except the incubation of recombinant VAR2CSA fragments.

Data availability

The atomic coordinates and electron microscopy data have been deposited in the RCSB Protein Data Bank and Electron Microscopy Data Bank under the following entries: 3D7 VAR2CSA ectodomain (PDB-ID 7FAS, EMD-31505), 3D7 VAR2CSA-CSA complex (PDB-ID 7FAP, EMD-31504).

Supplementary Figures

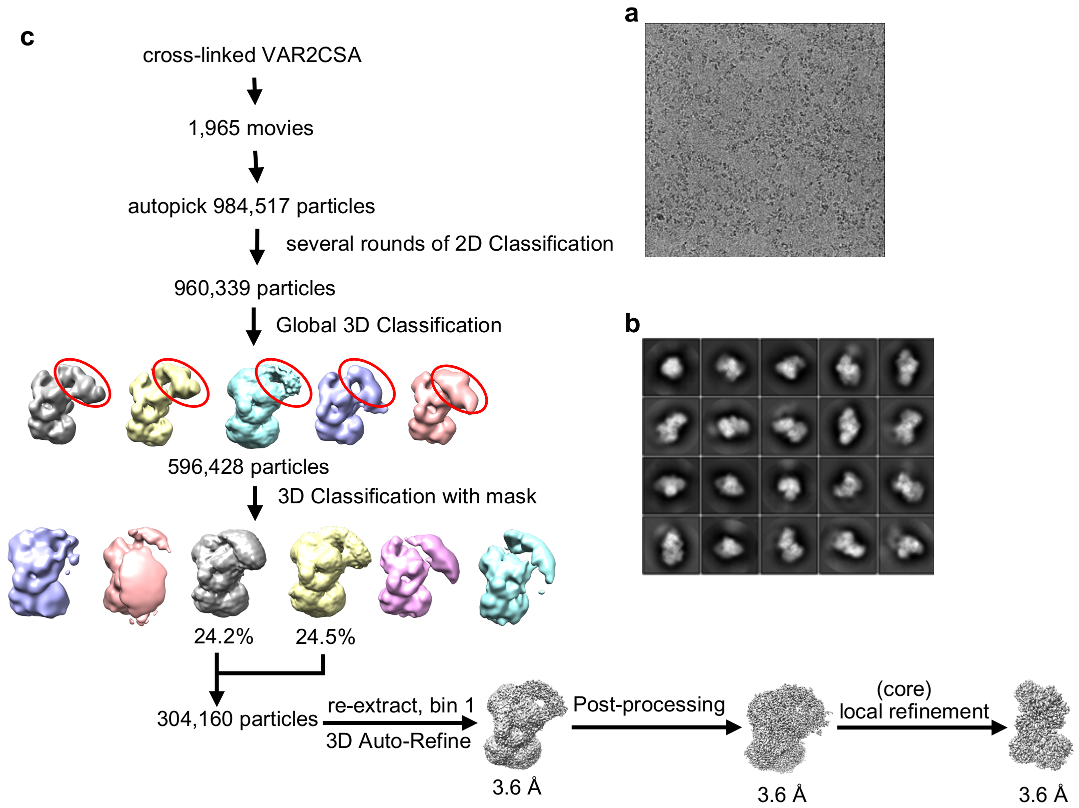

Fig.S1 Data collection and image processing of VAR2CSA ectodomain. a Representative image of cryo-EM dataset. **b** 2D classification result. **c** The flowchart of the cryo-EM image processing and 3D reconstruction for cross-linked VAR2CSA ectodomain. The highly flexible wing region highlighted in red oval in global 3D classification.

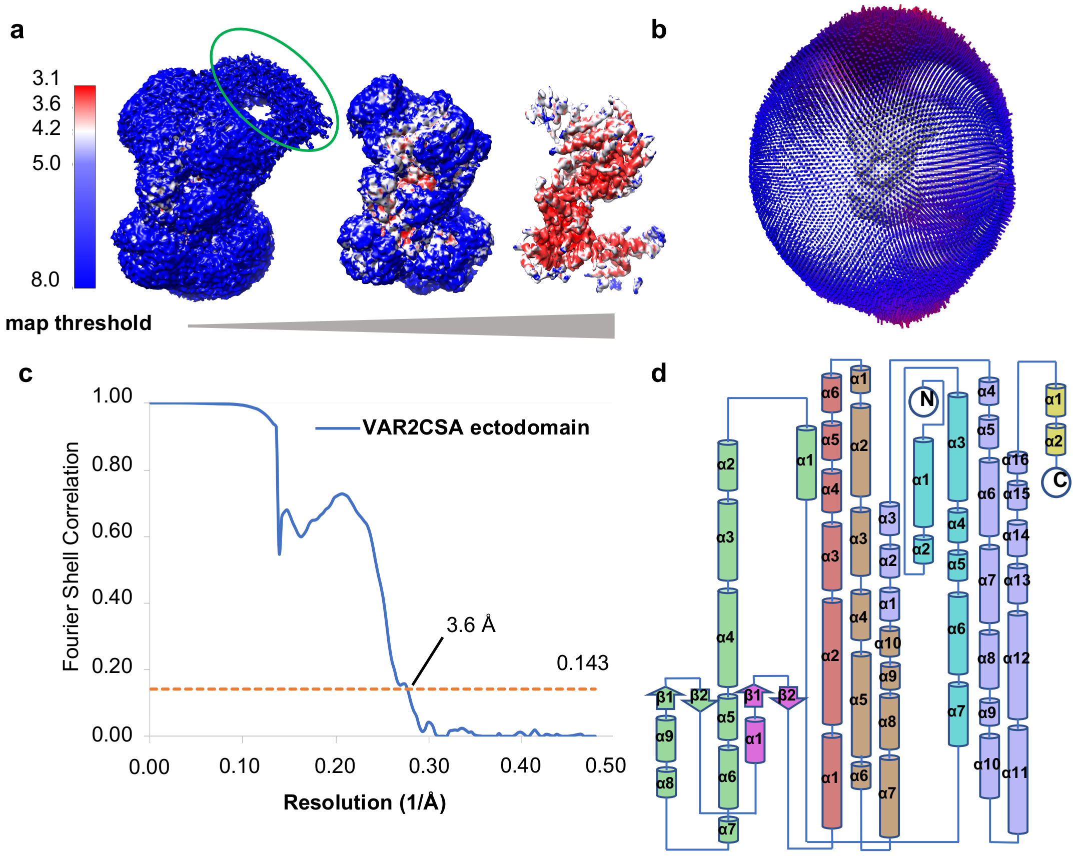

Fig.S2 Cryo-EM analysis of VAR2CSA ectodomain. a Local resolution of VAR2CSA ectodomain under different threshold of cryo-EM density map estimated by ResMap. Flexible wing region highlighted in red oval. **b** Angular distribution of cryo-EM samples of VAR2CSA ectodomain. **c** Gold standard-FSC curve of the refined map. **d** The topology of apo VAR2CSA ectodomain core region. α-Helixes shown as cylinders; β-strands shown as arrows.

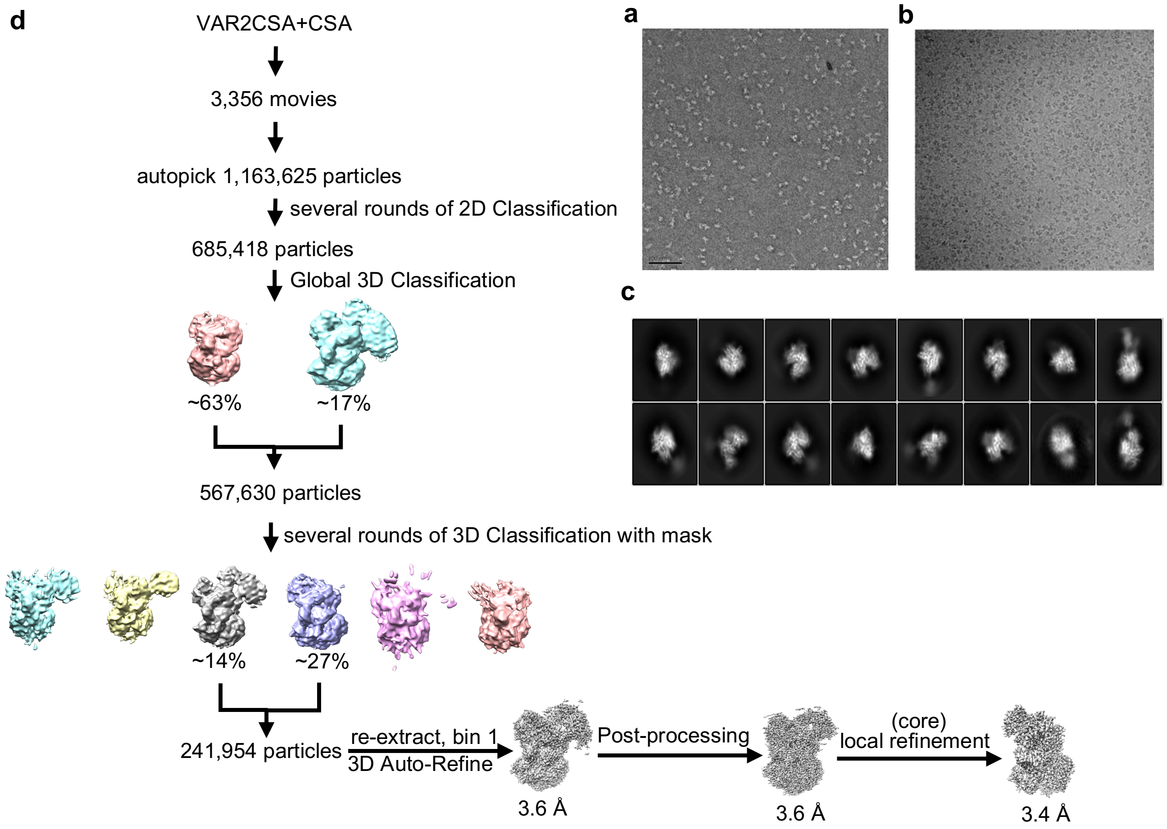

Fig.S3 Data collection and image processing of VAR2CSA-CSA. a-c Representative images of negative staining (**a**), cryo-EM dataset (**b**), and 2D classification (**c**). **d** Flowchart of the cryo-EM image processing and 3D reconstruction for VAR2CSA-CSA.

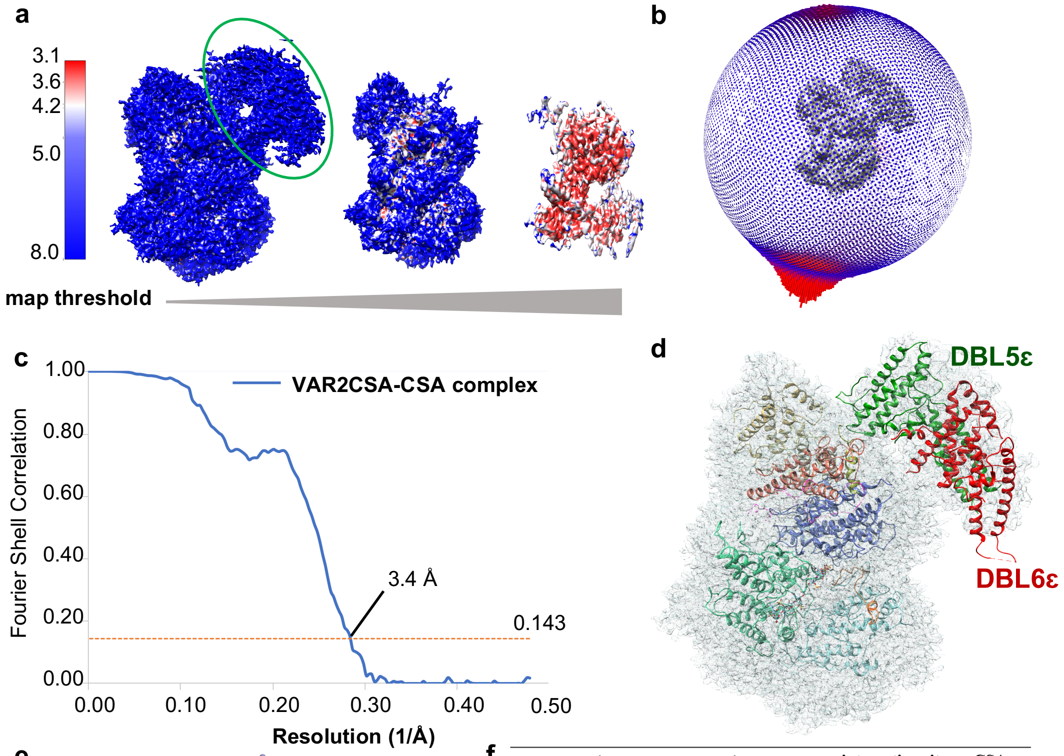

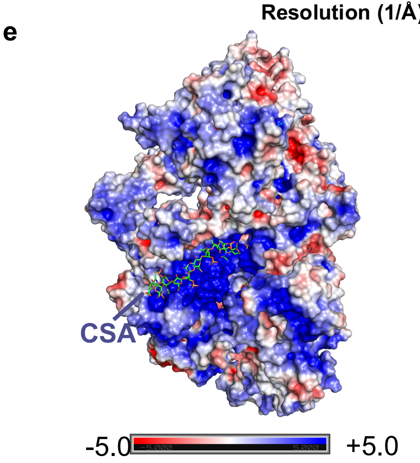

f

Domain	Residue	interaction site on CSA
NTS	Y44	ASG-2
	Y45	BDP-1
	N49	BDP-1/ASG-2
DBL2X	N557	ASG-12
	K561	ASG-10
	K562	ASG-8
	N576	ASG-8
	K828	ASG-8
	R829	BDP-9
	Q832	BDP-7
	K835	ASG-4
	R846	BDP-1
DBL4ε	E1880	ASG-4
	K1889	ASG-4/BDP-5
	R1890	BDP-3
	Y1899	BDP-1

Fig.S4 Cryo-EM analysis of VAR2CSA-CSA. a Local resolution of VAR2CSA-CSA under different threshold of cryo-EM density map estimated by ResMap. Flexible wing region highlighted in green oval. **b** Angular distribution of cryo-EM samples of VAR2CSA-CSA. **c** Gold standard-FSC curve of the refined map. **d** Density map of VAR2CSA-CSA with final structure model represented by ribbon diagram. **e** The surface electrostatic potential map of VAR2CSA-

CSA core region. CSA was drawn as sticks. **f** The key residues in VAR2CSA ectodomain responsible for interacting with CSA.

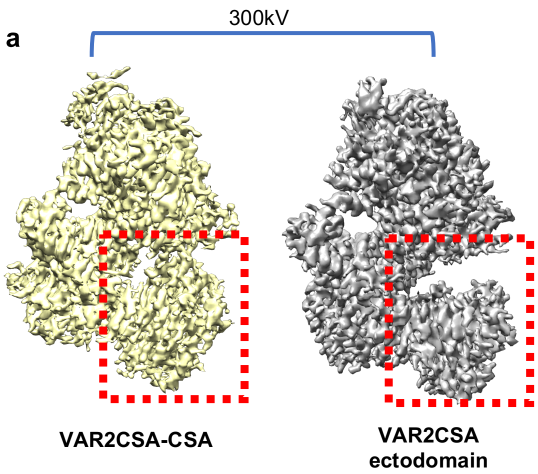

Fig.S5 Cryo-EM density maps represent the conformational change of VAR2CSA ectodomain upon CSA binding. Cryo-EM density maps reconstructed from 300 kV cryo-EM dataset (**a**) and 200 kV cryo-EM dataset (**b**) both displayed major differences highlighted within red dotted rectangles. VAR2CSA-CSA were colored in yellow and VAR2CSA ectodomain in grey.

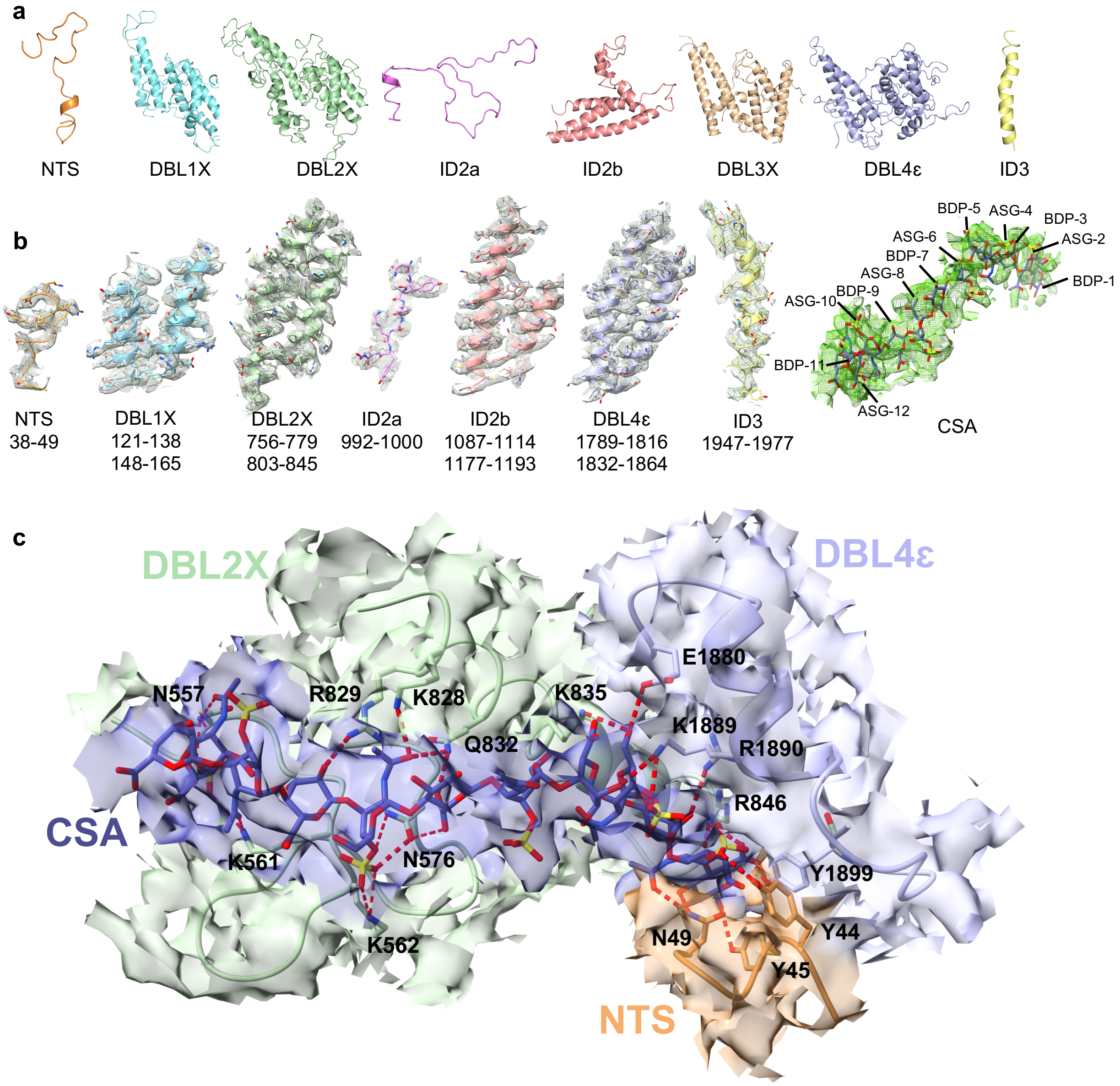

Fig.S6 Model composition and map density validation for VAR2CSA-CSA. a Ribbon diagrams of individual domains in the structure of VAR2CSA-CSA core region. **b** Representative cryo-EM density maps from VAR2CSA-CSA filled with structure models with side chains as sticks. **c** Interactions between VAR2CSA and CSA in the binding pocket were shown in cryo-EM map density and atomic models. Interacting residues from NTS, DBL2X, and DBL4ε were highlighted individually and hydrogen bonds were shown as red dashed lines.

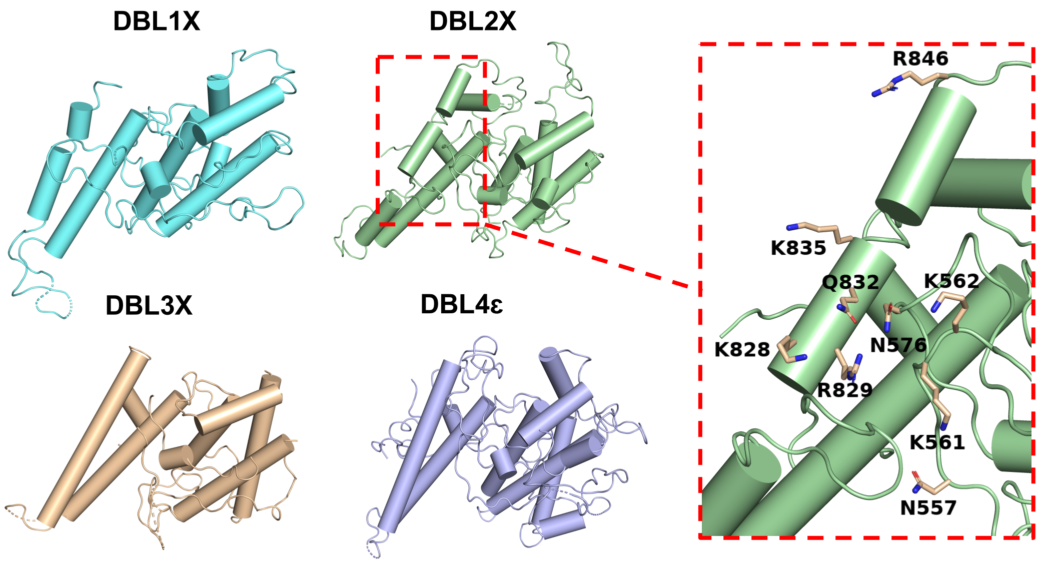

Fig.S7 The structural comparison between four DBL domains in core region. The overall structures of four individual DBL domains of the core region were shown. The nine CSA binding residues in DBL2X were highlighted, with side chains shown as sticks.

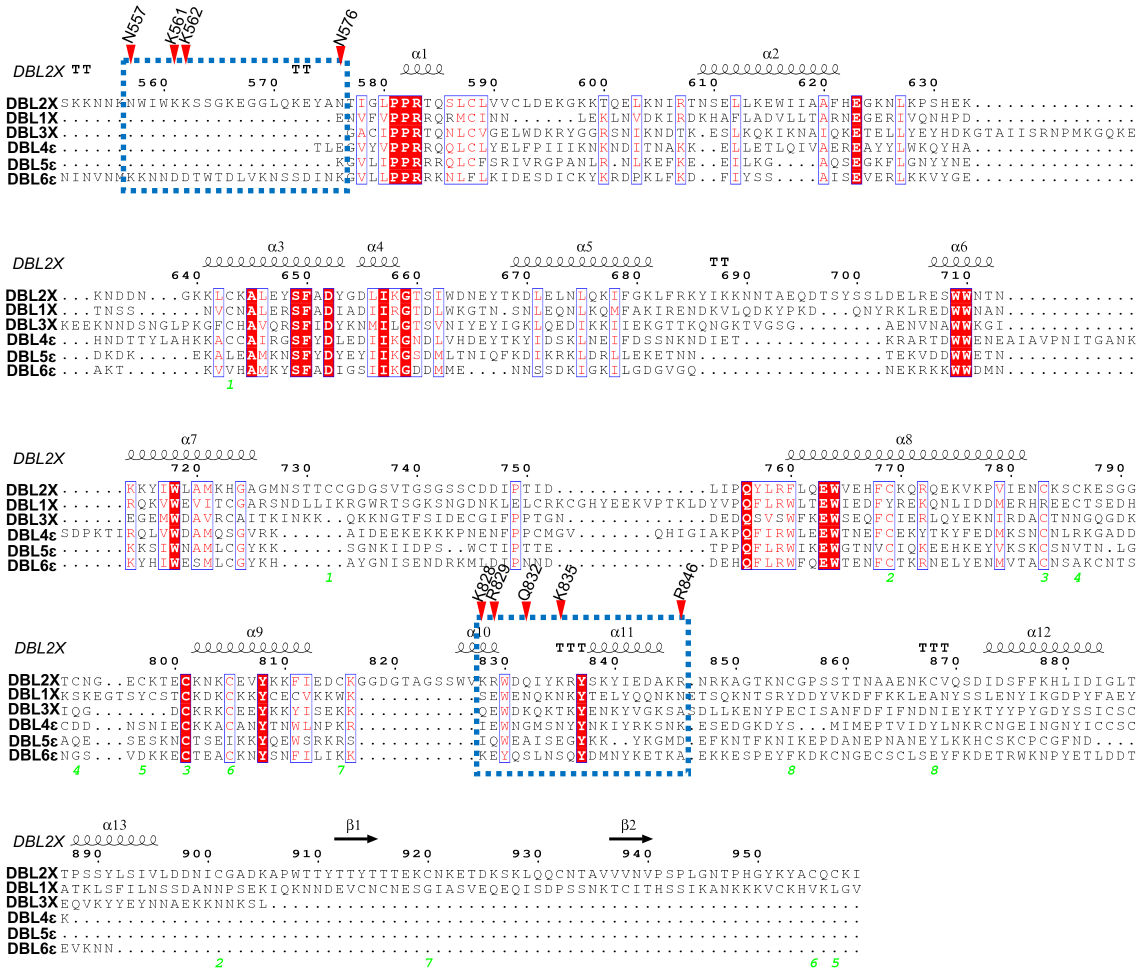

Fig.S8 Sequence alignment of six individual VAR2CSA DBL domain. The nine key residues of DBL2X involving in CSA binding were labeled and indicated by red triangles within the blue dotted rectangles. Secondary structures were labeled according to the structure of DBL2X using Espript3 online server.

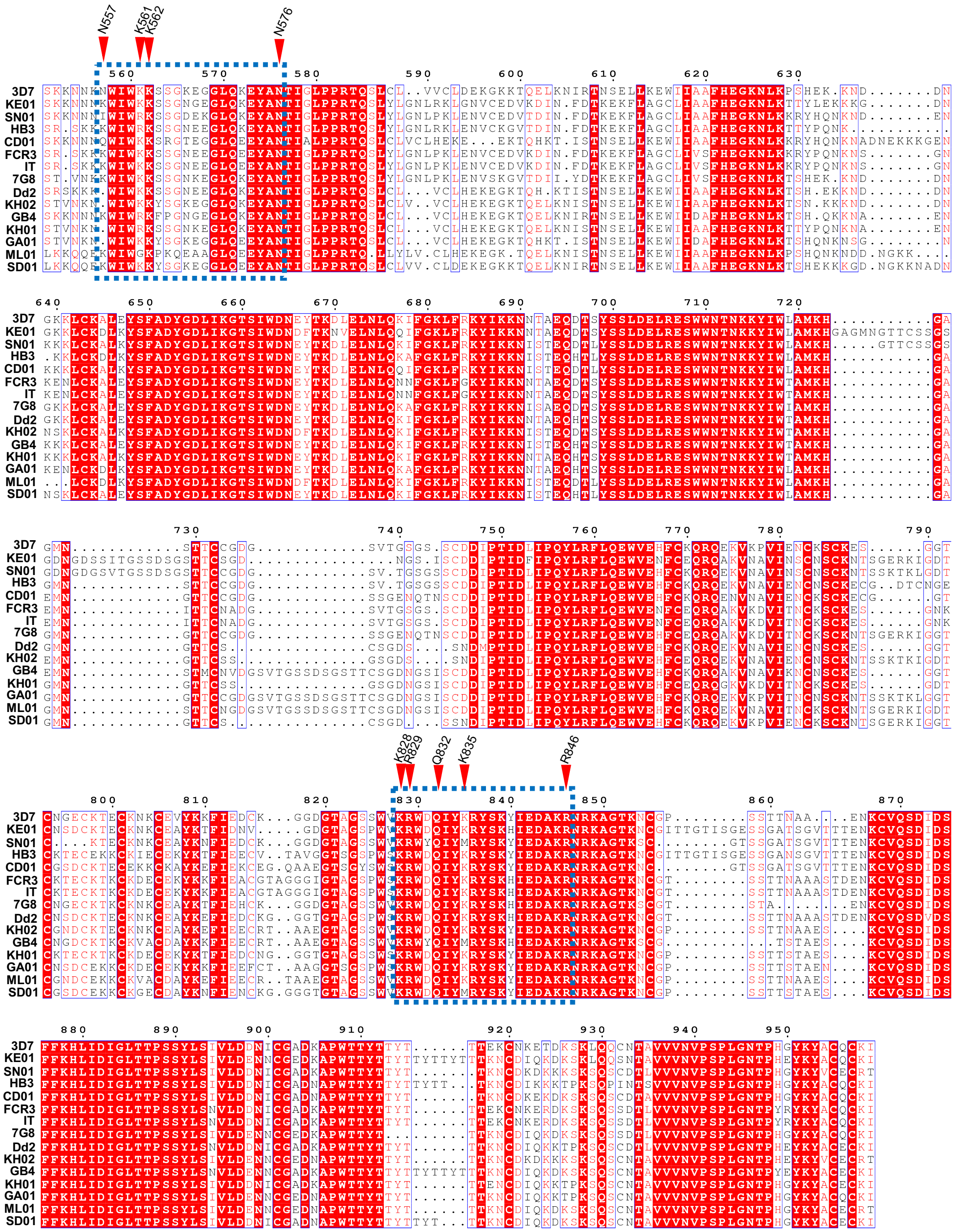

Fig.S9 The sequence alignment of VAR2CSA DBL2X domains from various *Plasmodium falciparum* species. Nine key residues for binding CSA were highlighted by red triangles within the blue dotted rectangles.

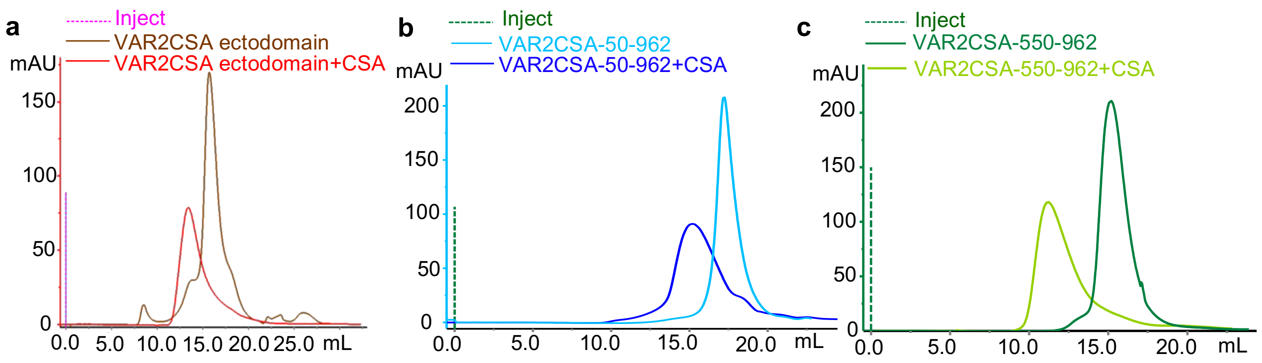

Fig.S10 Chromatogram comparison of gel filtration of various VAR2CSA fragments in the presence or absence of CSA. a VAR2CSA ectodomain on a Superose 6 increase 10/300 GL column. **b** VAR2CSA-50-962 (including DBL1X and DBL2X) on a Superose 6 Increase 10/300 GL column. **c** VAR2CSA-550-962 (DBL2X) on a Superdex75 10/300 GL column.

Table S1. Statistics of cryo-EM data collection and refinements

	Cross-linked VAR2CSA	VAR2CSA-CSA
Data collection and processing		
Magnification	130,000x	130,000x
Voltage (kV)	300	300
Electron exposure (e^- /pix/s)	~8.0	~8.0
Exposure rate (e^- /Å^2)	1.468	1.468
Number of frames per movie	36	36
Energy filter slit width (eV)	20	20
Automation software	SerialEM	SerialEM
Defocus range (μm)	-1.2~-2.2	-1.2~-2.2
Pixel size (Å)	1.044	1.044
Symmetry imposed	C1	C1
Micrographs (no.)	1,965	3,356
Total of extracted particles (no.)	984,517	1,163,625
Total of refined particles (no.)	304,160	241,954
Map resolution (Å)		
FSC threshold	0.143	0.143
Map resolution range (Å)	3.6	3.4
Refinement		
Map sharpening B-factor (Å^2)	-85.59	-123.93
Model composition		
Nonhydrogen atoms	10,173	10,057
Protein residues	1,229	1,209
Ligands	-	
B factors (Å^2)		
Protein	-176.93	-144.67
Ligand		
R.M.S. deviations		
Bond lengths (Å)	0.002	0.003
Bond angles (°)	0.517	0.585
Validation		
MolProbity score	1.76	1.75
Clashscore	8.54	9.83
Rotamer outliers (%)	0	0
Ramachandran plot statistics		
Favored (%)	95.68	96.48
Allowed (%)	4.32	3.52
Disallowed (%)	0	0

Movie S1.

The conformational changes of the core region of VAR2CSA ectodomain induced by recognizing and binding to CSA.

Reference

1 Kastner, B. *et al.* GraFix: sample preparation for single-particle electron cryomicroscopy. *Nature Methods* **5**, 53-55 (2008).

2 Mastronarde, D. N. Automated electron microscope tomography using robust prediction of specimen movements. *Journal of Structural Biology* **152**, 36-51 (2005).

3 Zheng, S. Q. *et al.* MotionCor2: anisotropic correction of beam-induced motion for improved cryo-electron microscopy. *Nature Methods* **14**, 331-332 (2017).

4 Zhang, K. Gctf: Real-time CTF determination and correction. *Journal of Structural Biology* **193**, 1-12 (2016).

5 Scheres, S. H. W. RELION: Implementation of a Bayesian approach to cryo-EM structure determination. *Journal of Structural Biology* **180**, 519-530 (2012).

6 Scheres, S. H. W. & Chen, S. Prevention of overfitting in cryo-EM structure determination. *Nature Methods* **9**, 853-854 (2012).

7 Kucukelbir, A., Sigworth, F. J. & Tagare, H. D. Quantifying the local resolution of cryo-EM density maps. *Nature Methods* **11**, 63-65 (2014).

8 Afonine, P. V. *et al.* Towards automated crystallographic structure refinement with phenix.refine. *Acta Crystallogr D Biol Crystallogr* **68**, 352-367 (2012).

9 Brown, A. *et al.* Tools for macromolecular model building and refinement into electron cryo-microscopy reconstructions. *Acta Crystallogr D Biol Crystallogr* **71**, 136-153 (2015).

10 Pettersen, E. F. *et al.* UCSF Chimera—A visualization system for exploratory research and analysis. *Journal of Computational Chemistry* **25**, 1605-1612 (2004).

11 Goddard, T. D. *et al.* UCSF ChimeraX: Meeting modern challenges in visualization and analysis. *Protein Science* **27**, 14-25 (2018).

12 Schrodinger, LLC. *The PyMOL Molecular Graphics System, Version 1.8* (2015).
